# P02.138. Acupuncture and meditation for military veterans: patient satisfaction and self reported symptom reduction

**DOI:** 10.1186/1472-6882-12-S1-P194

**Published:** 2012-06-12

**Authors:** A Hull, M Reinhard, K McCoy, J Akhter, A Duncan, K Soltes, C Jecmen, K Berndtson

**Affiliations:** 1 Veterans Affairs, Washington D.C., USA

## Purpose

Post-deployment health care for military veterans presents challenges to existing treatment models. Currently, the Department of Veterans Affairs (VA) sees numerous veterans with chronic, difficult to treat, and medically unexplained symptoms including, but not limited to, fatigue, chronic pain, headaches, gastrointestinal distress, concentration difficulties, disturbed sleep, anxiety, depression, and posttraumatic stress. The VA is increasingly investigating complementary medicine and integrative health care as resources to enhance its provision of patient-centered, empirically-supported care. Since 2007, the VA's War Related Illness and Injury Study Center (WRIISC) in Washington, D.C. has offered outpatient acupuncture and yoga nidra® (meditation) clinics as a complement to standard care for veterans of any combat era.

## Methods

Anonymous self-report satisfaction questionnaires were administered periodically throughout the year to a random subset of veterans in the yoga nidra® (n=184) and acupuncture (n=130) WRIISC-DC clinics in 2010.

## Results

The acupuncture clinic provided a total of 649 full body and 890 group encounters in 2010. Survey respondents reported complete or partial improvement in symptoms (96%), good to excellent quality of care (99%), and 99% would recommend acupuncture to another veteran. The yoga nidra® clinic provided a total of 1,318 group encounters in 2010. Survey respondents reported complete or partial improvement in symptoms (95%), very good to excellent quality of care (96%), and 100% would recommend yoga nidra® to another veteran. Further analyses show self-reported improvement in specific symptom areas and trends in data based on combat era.

## Conclusion

Satisfaction data suggests that the vast majority of sampled veterans who received acupuncture and yoga nidra® were satisfied with care quality, noticed symptom improvement, and would recommend acupuncture and yoga nidra® to other veterans. These data support further programmatic research to examine the effectiveness of these modalities and how they may best integrate with existing post-deployment health care.

